# Surveying the quality of bereavement support within a service setting: A pilot study using cognitive interviewing with bereaved people

**DOI:** 10.1177/02692163251353012

**Published:** 2025-07-28

**Authors:** Maja Furlan de Brito, Lucy E Selman, Alexandra Coelho, Barbara Gomes

**Affiliations:** 1 Faculty of Medicine, University of Coimbra, Coimbra, Portugal; 2 Centre for Innovative Biomedicine and Biotechnology (CIBB), University of Coimbra, Coimbra, Portugal; 3 King’s College London, Cicely Saunders Institute of Palliative Care, Policy and Rehabilitation, London, UK; 4 Palliative and End of Life Care Research Group, Population Health Sciences, Bristol Medical School, University of Bristol, Bristol, UK; 5 PSYLAB, Instituto de Saúde Ambiental (ISAMB), Faculty of Medicine, University of Lisbon, Lisbon, Portugal; 6 ISPA – University Institute, APPsyCi - Applied Psychology Research Center Capabilities & Inclusion, Lisbon, Portugal

**Keywords:** Bereavement, palliative care, health care surveys, health services research

## Abstract

**Background:**

Provision of bereavement care is not standard practice in palliative care, meaning routinely collected data to understand support quality at a service level is scarce. Survey research can fill this gap.

**Aim:**

To pilot and refine a survey and the survey method to measure the quality and variations in bereavement support at a service level.

**Design:**

Cognitive interview study. In analysis we used a combination of reparative and descriptive cognitive interview approaches to ascertain measurement errors and any participation distress.

**Setting/participants:**

People bereaved due to cancer (13–19 months post death) were consecutively recruited via a hospital-based palliative care service.

**Results:**

Twenty bereaved people participated (parental bereavement 8/20, partner 7/20, sibling 3/20, adult child 2/20); median age 59 (range 28–76), 6 men. Cancer deaths occurred 16–19 months before the interview. Identified measurement errors, mostly related to comprehension, were fixed. We refined the definition of bereavement support in the context of the survey and reordered the sections to reduce emotional burden and improve time completion. Participating in the survey was considered distressing yet personally valuable, relevant and important for improving bereavement support.

**Conclusions:**

We developed, piloted and refined a survey to assess quality and variations in bereavement support which can be used by palliative care services in clinical practice for quality improvement. Survey participation, while potentially distressing, was acceptable to and valued by bereaved people.

**What is already known about this topic?**

- Surveying about loss and bereavement support is challenging due to the topic’s sensitivity.
- Cognitive interviewing studies primarily focus on identifying errors by examining respondents’ cognitive processes. However, they often overlook a broader understanding of how the survey functions as a whole and its meaning to the participants.
- Robust survey tools are needed to help ensure palliative care services are bereavement-centered and the quality of bereavement support continuously improves.

**What this paper adds?**

- We developed a bereaved-conscious service-level survey using cognitive interviewing methodology with bereaved people to refine items and ensure acceptability.
- By combining different approaches to cognitive interview, we uncovered how essential concepts such as grief, caregiving burden, and bereavement support may be understood differently between participants and researchers.
- Order of the survey sections matter. With morbidity-first structure (followed by patient-care and support sections) we reduced time of completion and emotional burden for the participants.

**Implications for practice, theory, or policy**

- Inviting bereaved people to provide their views and feedback via a survey can directly shape the support provided by bereavement services, while also allowing bereaved people to reflect on their experiences, potentially assisting with meaning-making.
- If administered systematically, surveys can help identify gaps in support, not only in practical or logistic terms, but also cultural and emotional. Survey data can also help to demonstrate the value of a service, internally to the institution where it is offered, as well as externally, that is, to funders.
- Survey results can help position bereavement care as an integral part of health and social care, rather than an optional extra, leading to continuity of care for the bereaved.

## Introduction

Bereavement care is a crucial element of palliative care, but its provision is not yet standard practice^1^ and it is often under-resourced.^2^ Aware of this limitation, more palliative care services are considering expanding the bereavement care they offer.^1^ To ensure good quality bereavement support is routinely provided, we need to better understand variations in access to and outcomes of bereavement support and develop methods to measure the quality of the support provided. By systematically surveying the bereaved individuals within a service, we can reveal variations in access to bereavement support by capturing individual-level experiences across demographic (age, sex), relational (kinship, proximity/quality of relationship, availability of close relationships after death), and contextual factors (patient care, communication, awareness of available support). Survey administration enables identification of who accesses the support, who does not, and why, offering insights into potential barriers (stigma, lack of information, timing) and facilitators (proactive offers of help, prior connection to the service). By collecting data in a structured way within a single service, the study can identify within-service inequalities or inconsistencies, helping to inform more equitable, targeted, and responsive bereavement care provision. By collecting it in more than one service, this approach may help uncover systemic differences in support availability, such as variations in service outreach, staffing, connectedness to community resources, cultural competence, resources. This will help ensure palliative care services are bereavement-centric and the quality of support continuously improves.

Survey research is an appropriate method to tap into the needs, opinions and attitudes about bereavement support. It is a recommended method to gain insights into the quality of end of life care^3^ and a recognized way of capturing service users’ perspectives on care to inform quality improvements.^3,4^

Surveying about bereavement support is challenging due to the sensitivity of the topic^5^ and the difficulty of recruiting people who do not seek bereavement support but are likely to benefit from it.^6,7^ Thus, sampling considerations and the minimization of the burden and the maximization of the benefits that may be derived from participation are paramount in bereavement support research. Testing and refining a survey is needed to not only identify and understand potential response errors but also ascertaining the acceptability of the survey methodology, the usability of sensitive questions, the utility and relevance of the survey, and the objective and subjective response burden.^8^

In developing a robust survey, it is essential to identify any cognitive problems people experience when reading and responding to items to determine the response error.^9^ “Cognitive problem” refers to a mental hurdle during the process a respondent goes through to answer a survey question. This may be related to comprehension of the instructions, wording of the question, recalling the information needed to respond, or choosing the answer that most reflects one’s experience. “Response error” refers to a mistaken or inaccurate answer caused by a cognitive problem and resulting in an inaccuracy or anomaly that affects data quality. In Box 1 we illustrate this with an example. By understanding cognitive problems, we aim to reduce response errors via improved question design, pre-testing or clarification.

**Box 1.** Example of cognitive problem and response error applied to a bereavement survey.

The following example illustrates the difference between a cognitive problem and a response error in survey research: - Suppose a researcher asks: “*Have you received any formal bereavement support?*” The term “*formal*” may be ambiguous, as bereaved individuals could interpret it differently. One respondent might be unsure whether “*formal*” includes support groups, counseling sessions with a psychologist, or support made available through a church. - This reflects a cognitive problem, i.e. difficulty understanding the question. Although the respondent has been attending grief counseling offered for free by their church, they answer “*No*” because they do not consider this support “*formal*” due to its religious setting and because it is offered free of charge. This constitutes a response error, i.e. an inaccurate answer based on misinterpretation.

Tourangeau’s cognitive model of the survey response,^10,11^ and Conrad and Blair’s classification of problems^12^ are foundational models of survey methodology. Both models help design and test survey items. Tourangeau’s model has previously been applied in palliative care research with bereaved individuals.^13^ The author proposed a four-stage model that describes mental processes involved in responding to a survey. These are comprehension, retrieval of information, judgment, and response mapping. In Box 2 we provide examples of these, as applied to bereavement-focused surveys.

**Box 2.** Four stages of Tourangeau’s cognitive model of the survey response^10,11^ applied to bereavement survey.

- In a bereavement survey, respondents may interpret inconsistently key terms used in the instructions or questions such as “support,” “coping” or “grief” (comprehension); they may struggle to recall their experiences over long or emotionally complex periods (retrieval); experience difficulty evaluating the adequacy or impact of the support received (judgment); or find their experiences do not map easily onto the available response options (response mapping). - By probing the person to narrate their thoughts about questions, items and responses, while or after completing a survey helps anticipate erroneous answers or questions left unanswered (missing data). The following questions are helpful here: ○ Will the respondents understand the question/item (comprehension)? ○ Can they remember the information needed to answer (retrieval)? ○ Can the respondent judge the adequacy of the information recalled (judgment)? ○ Can they pick a suitable answer when they compare their experience to the response options provided (response mapping)? Examples we observed for each stage from our pilot study are presented in the Supplemental Material.

However, these frameworks do not examine the meaning of the survey questions from participants’ perspectives. Other models elucidate the degree to which the survey structure and content, in participants’ eyes, meet requirements. Examples are the O’Brien et al.’ coding system,^14^ based on Feinstein’s Sensibility framework,^15^ which takes a interpretivist perspective,^16^ acknowledging participants’ specific context, or culture. Taking this approach allows for exploration of the bereaved individual’s personal, cultural, and social experiences of grief and how the questions in the survey are meaningful and relevant to the respondent’s lived realities. By acknowledging participant’s unique contexts, we can identify potential sources of misunderstanding, cultural misalignment or emotional dissonance in the survey content. This in turn enhances the content validity of the tool, as it captures experiences of bereavement support in a way that is both sensitive and appropriate across diverse populations.

We developed a survey that is completed by bereaved caregivers 13 months after the death of a family member who died of cancer. It assesses experiences of informal and formal bereavement support, with the focus on service access and helpfulness, providing a comprehensive picture of a bereaved individual’s support network. We aimed to pilot and refine a survey and its method, testing it for response errors; relevance and practicality (overall impression and format, face and content validity, ease of use, and recall of episodes or events); acceptability; and the impact of taking part on bereaved participants.

## Methods

### Study design

This was a pilot study using cognitive interviewing.^9^ Cognitive interviewing is a method of collecting verbal information from participants on various aspects of a survey through the think-aloud method (verbalizing the thoughts when responding to a survey item or question) and probing (follow-up questions to encourage participants to elaborate on their thoughts, clarify responses, or provide deeper insights into their reasoning).^17^ It aims to externalize how participants perceive and interpret survey questions^12^ with a view to minimizing cognitive biases and improving accuracy, that is, the quality of survey data.

In research on sensitive subjects, cognitive interviewing is essential not only to identify potential response errors but also to determine a survey’s acceptability or appropriateness, relevance, practicality and usability,^8^ and subjective response burden.^5^

Recognizing the sensitivity of the topic, in choosing cognitive interviewing features, we were guided by protecting the bereaved people well-being while participating. Following previous reports on cognitive interviewing,^8,18,19^ we used spontaneous retrospective verbal probing to elicit understanding and function of the different survey sections as this method is considered easier, more comfortable, and less burdensome for participants.^18,19^ In comparison to the think-aloud method, it requires less time and may lessen the risk of triggering thinking about difficult experiences.^19^ A retrospective approach is also closer to normal conditions, permits a flow similar to a natural conversation, provides a global focus on the questionnaire, and does not result in a reactivity effect^18^ (changing of behavior of the respondents due to extensive probing after each item). We used mostly spontaneous probes as they are less burdensome for the bereaved, although they require more attention from the interviewer.

### Sample and setting

We aimed to recruit 15–20 bereaved relatives,^9^ consecutively identified through hospital records by the patient’s date of the death.

This service-based study took place in a general hospital in a metropolitan area in Portugal. The participants were recruited from records of the hospital’s palliative care service. Inclusion criteria: 13–19 months into bereavement, main family carers of the deceased person with cancer, the deceased/the family carer 18 years or older at the time of death, contactable, and live in the Metropolitan Area of Lisbon. Exclusion criteria: not speaking Portuguese, cognitive impairment that did not permit understanding of the informed consent and/or study materials, too burdened by the loss of their family member to be able to participate in the study.

### Measurements

#### Survey development

The survey development process passed through eight phases, including: (1) identifying major topics to be surveyed, (2) identifying variables within each domain to respond to research question, (3) identifying and prioritizing items for inclusion in the survey, (4) sequencing of the sections and development of instructions, (5) consulting with representatives of the bereaved (pre-piloting), (6) constructing the pilot version of the survey, (7) piloting the survey with cognitive interview, and (8) finalizing the survey. In its current form it contains 79 items, divided into 5 sections. More information in Supplemental Material.

#### Conceptualization of bereavement support

Bereavement support in this study refers to support provided to individuals before and after the loss of someone significant in their life. It aims to address emotional, social, informational, and practical/instrumental needs^20^ related to loss throughout the trajectory of bereavement,^1^ starting pre-death (anticipatory or pre-death grief^21^). It encompasses informal and formal support,^22^ with formal support understood to refer to three levels^23,24^: provision of information, community-based formal and organized service provision, and professional therapeutic services (including specialized therapeutic services).^25^

In the survey (included in full in Supplemental Material), bereavement support is measured with questions like “Have you talked to any of the following to discuss how you have been feeling or what you have been thinking about the loss of your [family member]?” and “Have you received, used or participated in any of the following types of support?” with answer options: Yes; No, but would have liked to/have considered it; No. These two questions are followed by a list of sources of support. To capture the informal and formal level 1 support we ask about receiving support from: (1) different sources, such as closest informal network (family, friends, pets), wider social network (family doctor, pharmacist, hairdresser, social media), and (2) types of support, such as receiving a leaflet with basic information, having the opportunity to be alone with the deceased after the death, receiving a condolescence letter, bereavement call/visit by the clinical team. Formal level 2 support was captured by asking about: (1) support received by the patient’s health care team, complementary therapy, and (2) types of support such as self-help groups, activity-based programs for the bereaved individuals. To capture specialized support we asked about contacts with psychologist and/or psychiatrist.

Both questions include the possibility to add contacts and types of support that are not amongst the pre-defined options. The survey also asks “How helpful was this support?” (Likert scale from 0 (not at all) to 5 (extremely), and whether the support was used before and after the death.

#### Other variables

The survey included a combination of items used in previous studies alongside standardized items developed specifically for the study. The structure and the number of items per sections is available in the Supplemental Material. The section on bereavement outcomes includes the Prolonged Grief Disorder (PG-13).^26,27^ PG-13 is a well-established 13 item questionnaire that assesses symptoms of prolonged grief disorder. Ten of the items are scored on a Likert scale (1-not at all; 5-overwhelmingly) to indicate how often that item describes how the bereaved is feeling. The following three items assess whether the bereaved had lost a significant other, how long ago the death occurred, and the impairment associated with symptoms of grief. Presence of prolonged grief disorder, however, needs to be further explored and confirmed in a clinical interview. The PG-13 has been psychometrically validated for the Portuguese population, demonstrating a Cronbach alpha of 0.932.^27^

The section on prior caregiving experience and healthcare includes questions used in the QUALYCARE study, a population-based mortality follow-back survey previously used in the UK and the US.^28^ To assess subjective perception of the caregiving burden we used one item of the Zarit Burden Interview (ZBI-22) retrospectively (“*Overall, how burdened did you feel in caring for your relative?*”; 0 = never to 4 = nearly always).^29^ Other sections include items developed for the purpose of this survey. A description of the development and structure of the survey questionnaire is in Supplemental Material.

### Analysis

We used a combination of reparative and descriptive approaches to analyze the generated cognitive interviewing data.^18^ We first sought to determine errors to improve the survey and consequently improve the quality of the data (reparative approach). We added the descriptive approach to understand the variety of errors and all major sources of distress from the survey for the bereaved. Aligned with this, we used two different analytical models: applying pre-existing set of codes to the data (deductive coding based on the Tourangeau’s cognitive model and Feinstein’s Sensibility framework, and inductive coding based on patterns and themes identified directly from the data).^18^ This combination of approaches is more aligned with the nature of grief and bereavement, with results reflecting the perspectives and perceptions of the participants.

To determine errors we used top down (deductive) cognitive coding^18^ based on Tourangeau’s four stage model of respondent’s cognitive processes (comprehension, retrieval, estimation, response formulation; see examples in Supplemental Material).^10,11^ We organized additional feedback by Feinstein’s Sensibility framework (overall impression, face and content validity, ease of use, response options, format, episodic component; see examples in Supplemental Material).^15^ Both models provide a predefined set of codes that capture potential issues related to a specific item, section or the survey as a whole. The impact of taking part in the survey was explored inductively. We used pattern coding,^18^ to explore whether cognitive processes and survey experiences differed depending on the intensity of grief felt by participants. Matrix queries in NVivo (cross-tabulation of the data set to intersect experiences and intensity of grief to look for patterns) were used to explore and identify patterns.

Data were original verbalizations and researcher’s notes. These included observations made during and after the interview. All interviews were recorded, transcribed verbatim and analyzed in NVivo (v14).

### Ethics

The study was approved by the ethics committees of the Faculty of Medicine of the University of Coimbra (CE-154/2019) and of the Local Health Care Unit Santa Maria responsible for the hospital where the study took place (405/21). All participants provided informed consent. We followed a distress protocol to identify and handle participant’s distress. This included referral pathways in case of expressed need for bereavement support during the interview or if the distress was triggered by the survey participation.

## Results

### Sample characteristics

**Figure 1.**
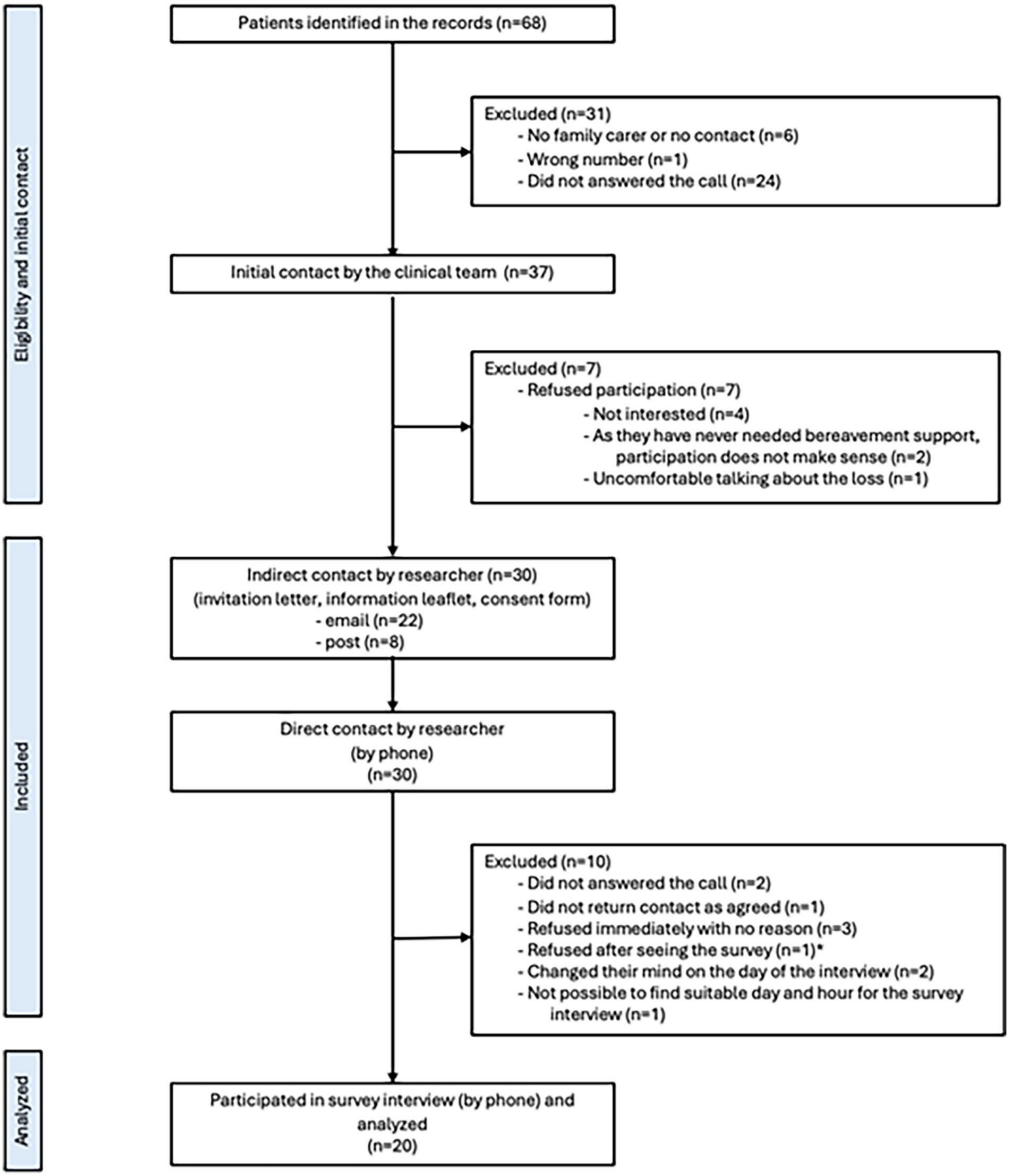
Participant recruitment flow. *Note*. *The participant asked to view the survey beforehand to help decide on participation.

We interviewed 20 participants (6 men) with median age 59 years (ranging 28–76). Level of education varied from primary school or less (*n* = 4), secondary school (*n* = 9) to under/post-graduate level (*n* = 7). A variety of losses were included: parental (*n* = 8), partner (*n* = 7), sibling (*n* = 3), and adult child (*n* = 2), occurring 16–18 months before. Interviews (median length 39 min, ranging 31–81 min) were conducted over the phone (preferred option by the bereaved), with 4 participants deciding to have the phone interview in a public space (park, café). Seven had experience of formal bereavement support and four expressed high need but had not received/sought formal support. The remaining received support from family in friends, with different levels of satisfaction. Figure 1 shows the recruitment flow of participants with reasons for non-participation when provided. Response rate is reported in Supplemental Material.

### Cognitive processes

**Figure 2.**
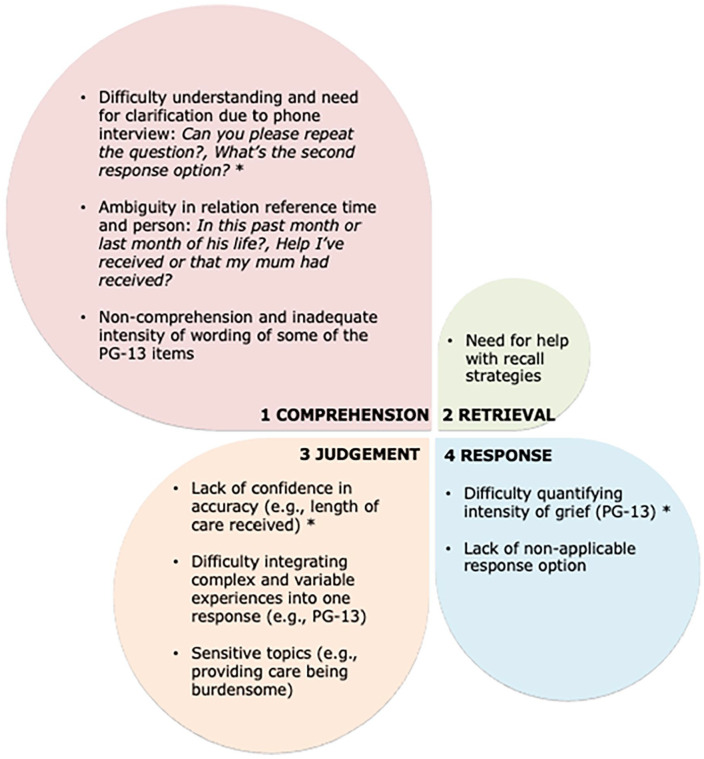
Difficulties in responding to survey questions by Tourangeau’s four stage model of respondent’s cognitive processes.^10,11^ *Note*. The size of the petals corresponds to the frequency of the problems identified in area. *The problem most frequently represented within each stage of response formulation.

Figure 2 summarizes the most common difficulties in responding to the survey. There were 52 occurrences coded under comprehension, 18 under judgment, 15 under response, and four as retrieval of information (four stages of Tourangeau’s model of respondent’s cognitive processes^10,11^). The comprehension stage was the most problematic due to the interviews being conducted over phone. The most problematic part of the survey proved to be the standardized measure of grief (PG-13), with occurrences of cognitive issues related to all four stages of response formulation. Table 1 presents refinement of the survey, and the method of administration based on the pilot. More detail is available in Supplemental Material (Tables 1–4).

**Table 1.** Refinements in survey method to improve response formulation.

Refinement of	Actions taken
Survey	• Addition of “non-applicable” response option. • The 1-item retrospective measure of subjective caregiving burden from ZBI-22 resulted in a floor effect (17/20 answered “*none*”). Participants commented that in their view the word “burden” does not apply to care at the end of life (*n* = 5) as “*we do this out of love*.” • PG-13^ref^ was the most problematic due to non-comprehension of words (*feeling stunned*, *emotionally numb*), intense wording (*stunned*), perceived inappropriateness of how it quantifies different aspects of grief. We prepared explanations of words in a more acceptable language.
Survey method	• Invite the participants to be in a quiet place with no distractions (while trying to meet their need for being in a particular place) • Signal clearly to participants the change in time/person focus of the questions. • Encourage use of recall strategies, i.e., help to reconstruct the past event/process.

### Sensibility

Analysis of survey’s sensibility by Feinstein’s criteria showed the survey is acceptable, meaningful, relevant and practical for the bereaved. Table 2 shows refinement of the survey, and the method of administration related to sensibility. A detailed description of results for each criterium is presented in Supplemental Material.

**Table 2.** Refinements in survey method to improve relevance, practicality, and acceptability.

Refinement of	Actions taken
Survey	• Participants pointed out PG-13′s inappropriate assumptions (face validity) about the constant rather than fluctuating nature of grief and struggled to quantify signs of grief in the “*linear*” manner the items required. We added introductory text to the grief related questions, acknowledging the fluctuating nature of grief and needs for support and use open ended questions to allow for additional comments and explanation of their experience. • The 1-item retrospective measure of subjective caregiving burden from ZBI-22 resulted in a floor effect (17/20 answered “*none*”). Participants commented that in their view the word “burden” does not apply to care at the end of life (*n* = 5) as “*we do this out of love*.” • Coding for content validity identified a possible mismatch between understanding what bereavement support entails in the context of the survey. While the researchers initially considered only emotional aspect of support, the bereaved people referred also to social, financial, and practical support. We prepared a working definition of bereavement support. • Exploring the ease of use resulted in the change of the order of the sections to reduce emotional burden and shorten completion time. Namely, from morbidity-last (informal and formal caregiving experience first, followed by grief-related needs and bereavement support) to morbidity-first structure (bereavement support and needs, followed by informal and formal caregiving experience).
Survey method	• Change in format of the question about barriers to support. This was reformulated in a parent-child type of question to reduce the number of response options for the ease of application over the phone. • A stronger emphasis to be put on the fact that the data collector is not a part of the clinical team to prevent bias.

### Impact of the survey and participation

**Figure 3.**
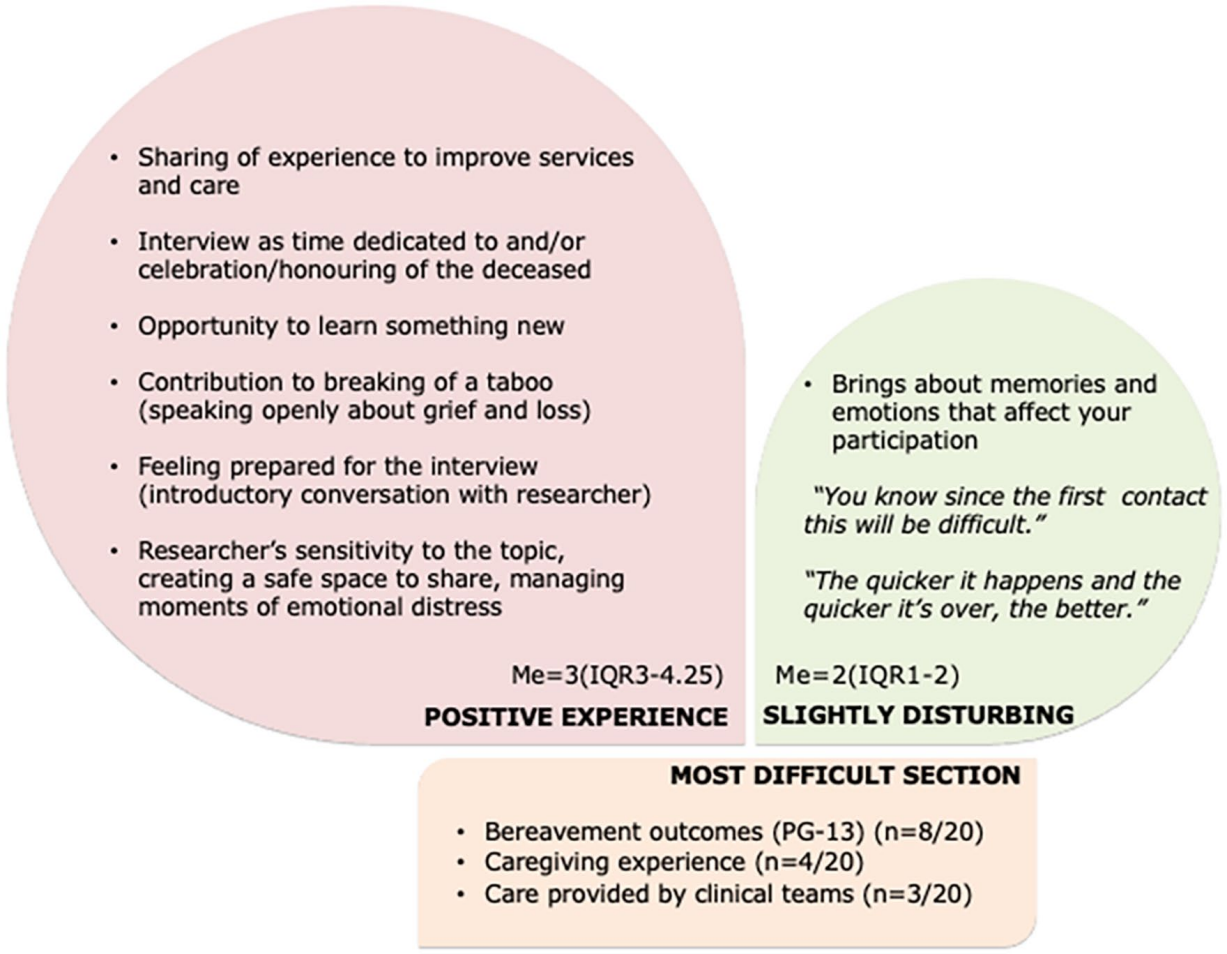
Experience of participating in a service-based bereavement support survey. *Note*. Me: median; IQR: interquartile interval.

More than half of the participants (*n* = 11) thought the interview and the questions were not at all/slightly disturbing and most (*n* = 18) found taking part in the survey a positive experience. Four people found the interview very distressing but reported it was simultaneously a very positive experience. Figure 3 summarizes the subjective experience of taking part in the pilot study. Emotionally, the most burdensome section was talking about the experience and intensity of grief (*n* = 8). The sections about providing care (formal and informal) were, jointly, difficult for seven of the bereaved. Due to an expressed need for formal support but not receiving it at the time of the interview, following our distress protocol the researcher referred three participants to the attending clinical psychologist from the palliative care team (with participant’s permission). Based on participants’ feedback, we refined procedures related to first contact, follow-up, and other interactions to ensure that taking part in the survey was as positive and beneficial experience as possible for all (Table 3).

**Table 3.** Refinements in survey method to improve experience of participating.

Refinement of	Actions taken
Survey method	• Contact the bereaved as soon as possible after being approached by the clinical team and accepting participation. • When scheduling the interview, have a conversation about expectations and worries with all participants. • Conduct a follow-up conversation if needed. • In the emotionally more burdensome section (experience of grief) indicate how many questions are left until the end.

### Is intensity of grief reflected in response error and experience of participation?

We hypothesized the experience of the survey may differ in terms of cognitive issues, meaningfulness of the survey, emotional burden, and experience with participation depending on the intensity of grief. We did not identify any differences in issues related to cognitive processes or meaningfulness of the survey. We did, however, observe a difference in the quantity of feedback received from the participants. While their comments did not differ in quality (number of issues raised, survey being more/less meaningful, experience being more/less burdensome), those who had lower intensity of grief gave more detailed feedback and shared longer narratives. Those with higher intensity of grief provided shorter, less detailed feedback on items and difficulties which with additional probing did not deepen. This may imply we should avoid additional or extensive probing with those with higher grief intensity as this a more withdrawn answers may be a coping strategy.

## Discussion

### Main findings

This cognitive interview study set out to test a survey and the method of application for measuring quality and variations in bereavement support at a service level and showed the proposed tool and method do not present a risk for major measurement errors, is comprehensive, meaningful and acceptable for 13–19 month-bereaved people.

Based on the experience and feedback we received from the bereaved the survey is meaningful, relevant and practical. Format-wise, it was felt easy to complete. Participation does come with emotional burden, but with an understanding and supportive approach from the researcher, this is balanced out with an overall positive experience of participating, and recognizing the survey’s value for improving support services. Cognitive difficulties mostly originated from the administration mode as comprehension was at times limited over the phone. The standardized measure of grief (PG-13) proved to be the most problematic section due to strong wording and emotional burden. Currently this is the only validated Portuguese tool for grief intensity, and this was the first time it was cognitively tested. Our experience has informed the design of the ongoing validation study for the revised PG-13.^30^ Also, we found that those with higher intensity of grief provided shorter, less detailed feedback on items and difficulties which with additional probing did not deepen.

### What this study adds

Response rates of bereavement support utilization studies range 13%–78%,^31–34^ however it is not always clear how rates are calculated. Ours falls withing the higher range of the expected, possibly due the use of a well-planned and sensitive approach that involved carefully worded contacts kept at a necessary minimum, yet reactive to participants’ needs (additional contacts, and availability of the researcher for conversation when requested). While a high response rate is preferable, it is not the only measure of quality.^35^ Our sampling strategy resulted in a varied sample in terms of sociodemographic characteristics, bereavement well-being, and types of losses, which increases the validity of the results and gives an optimistic perspective on future similar studies as it suggests it is possible to capture a diverse sample of bereaved with a good response rate and, thus maintain an acceptable level of non-response bias.

Although considered a fundamental part in survey testing, cognitive interviewing is commonly used in survey development or pilot testing of mortality followback surveys focused on the quality of end-of-life care.^3,4^ In bereavement-focused studies, the emphasis has been mostly on benefits and risks of participating in bereavement studies as a response to ethical considerations raised by ethics committies.^36^

In general, our finding aligns with previous studies where clarity and understanding were the major issues.^37,38^ Although using Tourangeau’s four stage model of respondent’s cognitive processes in our study was able to identify major errors in how the bereaved came to answer to the items, it was less helpful in capturing a more general understanding of how a survey functions as a measure of quality and variations in bereavement support. With a wider approach (looking at sensibility using Feinstein’s framework^15^) we were able to uncover further issues, crucial for aligning the understanding of the core concepts surveyed, such as grief, caregiving burden, and bereavement support, between participants and researchers. In this way we can better capture the different realities and variations in bereavement support as intended. Identifying that those who are experiencing stronger grief response may have more difficulty in engaging with a task that requires additional effort (providing feedback alongside or after responding to questions) tells us we potentially have less understanding of the difficulties they encounter with the survey. Also, given the lower engagement and less detailed answers, we may have missed relevant information for improving the survey for this sub-group. However, this preferential pattern of participation may reflect a greater need for privacy, which must be respected.

The need for reordering the sections to lessen emotional burden was identified through the sensibility analysis. This is a relevant issue since the order of sections seems to affect prevalence estimates of psychological measures in surveying the bereaved.^39^ This may have implications for the estimation of bereavement outcomes and needs for support, and consequently for the planning and organization of bereavement support services. Hauksdóttir et al.’s trial^39^ with 61 widowers compared two structures of a survey (morbidity-first vs morbidity-last), and found that participants who responded to questions on their morbidity after patient care-related questions obtained higher risk ratios on levels of depression and well-being. Attrition rates were not affected. With the order change in our survey moving morbidity to the beginning of the survey we expect to control for a potential cognitive bias in responses, where emotional recollections triggered by questions about caregiving experience and experience of communication with the clinical team may have a priming effect^35^ influencing the assessment of grief symptoms. Our pilot also showed that with a morbidity-first structure, we reduced time of completion and reduced emotional burden for the participants.

Existing studies have been criticized for failing to recognize the uniqueness of the grief process and to capture individual differences and cultural considerations in the way the bereaved engage with services and resources within and outside of their formal network.^40^ In our study, the bereaved people told us about their experience and understanding of grief in response to the items of standardized measures that did not always correspond to their view on grief. This is an important finding to take into account in future outcome measurement development. Notwithstanding, the inclusion of an additional free-text option or introduction of open questions may be a solution in making a survey more bereaved-centric. This addition may be understood as an approach tailored to the individual’s need for more reflection and storytelling, allowing for personal differences in the experience of loss to emerge.

### Recommendations for survey design and testing

We provide the following recommendations for designing and testing bereavement-conscious surveys that considers respondent well-being. We advise testing the survey for more than just response errors, exploring how the survey functions within a wider socio-cultural context that is particular to a service or community. We recommend adapting the level of probing to the individual. Although based on a small sample, our findings suggest those who have higher needs for support may in first instance need to protect their well-being; as researchers and clinicians we should support this need. Our experience confirms that placing patient-care related questions as the last section, or after assessment of the intensity of grief/need for support, ensures that the focus is on the bereaved person, and their needs first before asking the respondent to reflect on the experience of patient care. Adding space for free-text in addition to using standardized questions/scales allows for individualization of the survey, giving the participant the opportunity to comment on and express their grief and experience in their own terms. Debriefing after completion of a survey provides additional opportunity for the respondent to communicate aspects of their experience/views not captured elsewhere.

### Implications for bereavement practice, policy, and research

Study findings provide robust, empirical data on real-world experiences and unmet needs, including patterns, disparities and factors associated with support uptake and outcomes, contributing to the evidence base for evaluating bereavement interventions, informing theoretical frameworks, and shaping policy and service design. We found that participating in a survey could have therapeutic benefit for bereaved people, helping them feel their experiences are taken seriously and recognized, and, for some, contributing to meaning-making.

At a service level, the bereavement survey tested here can serve as a service evaluation and improvement tool, informing service delivery and development and resource allocation, helping to ensure the format and nature of bereavement support is tailored to population need. In clinical practice, systematically collected data may help increase appropriate and consistent referrals,^41^ potentially reducing the long waiting lists that exist in some areas. It might also help progress the development of core outcomes for bereavement support. If administered systematically, surveys can also help identify problems with access; demonstrate the value of a service, both internally and to funders and commissioners; and support better collaboration across settings and sectors. Ultimately, it can help to position bereavement care as an integral part of healthcare, supporting continuity of care for the bereaved.^1^

Before implementing the survey more widely, its performance should be explored in more diverse and larger bereaved populations, particularly those underrepresented in bereavement studies (e.g. minoritised ethnic communities, lower income, LGBTQ+). This will help assess and refine the survey’s cultural and contextual applicability and inclusivity. Future research should explore alternative formats or support strategies that can facilitate participation without increasing emotional burden. Testing the role of free-text or more narrative options in enhancing perceived relevance and emotional expression is also recommended, especially among those with higher levels of grief or who find standardized measures restrictive and misaligned with their experience. Finally, further research is needed to systematically investigate the role of surveys in reflective and meaning-making processes.

### Strengths and weaknesses

To our knowledge this is one of the first studies that cognitively tested a bereavement survey with a combination of reparative and descriptive approaches and drawing on existing models to ensure attention to cognitive and emotional processes. We did that by using different coding schemes to combine different foci, namely, cognitive processes and problems, the suitability of questions for the purpose and the context, and experiences of survey participation. However, the results do not tell us about to what extent this pattern will be present within a survey sample. Also, the process itself is somewhat different from the reality of a survey interview. The trade-off between more thorough techniques (think aloud method, simultaneous probing) and well-being of the bereaved may led to smaller reactivity effect for the price of unidentified aspects of measurement error.

## Conclusion

Our theoretically-based systematic analysis of the cognitive and emotional response processes and experiences of the survey proved helpful to unify the perspectives of the researchers and the participants on the subject matter, to ensure data collection prioritizes participants’ well-being, decreases risk of harm, and improves potential benefits of research participation. This comprehensive pilot testing promoted participant well-being as well as increasing confidence in our final version of the survey questionnaire and methodology for future use in routine practice for quality improvement, particularly by palliative care services. When administered in a systematic way, the survey contributes to equity and inclusivity as it helps to uncover diverse experiences and strengthens the overall evidence base for bereavement care.

## Supplemental Material

sj-docx-1-pmj-10.1177_02692163251353012 – Supplemental material for Surveying the quality of bereavement support within a service setting: A pilot study using cognitive interviewing with bereaved people Supplemental material, sj-docx-1-pmj-10.1177_02692163251353012 for Surveying the quality of bereavement support within a service setting: A pilot study using cognitive interviewing with bereaved people by Maja Furlan de Brito, Lucy E Selman, Alexandra Coelho and Barbara Gomes in Palliative Medicine
