# Supplementary material for: Surveying the quality of bereavement support within a service setting: A pilot study using cognitive interviewing with bereaved people
Source: Palliat Med. 2025 Jul 28;39(8):884–96. doi: 10.1177/02692163251353012 (PMC12405657; doi:10.1177/02692163251353012)
Supplement: sj-docx-1-pmj-10.1177_02692163251353012 – Supplemental material for Surveying the quality of bereavement support within a service setting: A pilot study using cognitive interviewing with bereaved people [file sj-docx-1-pmj-10.1177_02692163251353012.docx]

**Supplementary material**

***Response rate***

Of all records of eligible deceased patients, 10% (7/68) family carers were not contactable and 35% (24/68) did not answer the telephone call. 19% (7/37) refused participation in the first contact with the recruiting clinician. Only one person explicitly said this was due to not feeling comfortable responding to questions about the topic of loss and support. The remaining persons stated lack of interest or not having any need for support, therefore feeling it did not make sense to participate. In the second stage (contact with researcher), 10% (3/30) were not contactable and a further 20% (6/30) refused participation (no reason specified). Response rate was 29% (20/68), considering all identified eligible deceased as the denominator, 54% (20/37) if the denominator were all contacted family carers, and 81% (20/30) considering only those who gave permission to be contacted by the research team.

The researcher made at least one initial contact with the participants to review the objective of the study, explain the consent procedure and answer any additional questions. An additional preparatory conversation was held with six participants to discuss questions/ concerns and how to address the deceased during the interview. Two asked to see the survey beforehand, one of whom decided not to participate saying they thought their experience “*would not add much to the study*”.

**Analysis by the Cognitive model of the survey response process^1,2^**

Table 1. Definition and examples for Comprehension stage of the Cognitive model^1,2^

| **Comprehension** | |
| --- | --- |
| **Definition** | **Examples** |
| Understanding question intent and meaning of terms:   - Requests for clarification and signs of difficulty in understanding of wording, focus of the question or the question itself, and of instructions - What key terms do participants link to concepts they aim represent | *P: What do you mean by “emotionally numb”? What is this? What does it mean?*  *P: I can’t see exactly what you mean when you say “stunned”. What’s the purpose of this question?*  *P: Are you asking me about the support I had received or my mum?*  *R: Have you received support by a complementary therapy practitioner? For example, reiki or acupuncture.*  *P: Oh, I was thinking more of yoga when you mentioned complementary therapy.*  *R: Has it been difficult for you to accept the loss?*  *P: What does having “difficulty in accepting the loss” mean?* *I don't think so, but I mean, that's a bit vague, really.* |

*Note:* P=participant; R=researcher.

Table 2. Definition and examples for Retrieval stage of the Cognitive model

| **Retrieval** | |
| --- | --- |
| **Definition** | **Examples** |
| Retrieval of relevant information from memory:   - Type of information that needs to be recalled and how recallable it is - Recall strategy used | *R: Did you receive any information about bereavement support?*  *P: To be honest, I don’t remember.*  *R: How many hours per day, on average, did you dedicate to care of your sister?*  *P: I was at her house the whole day, from the morning till late evening.*  *R: Would that mean arriving around 9am?*  *P: Hmmm, actually later, because she liked to sleep in the morning. So, 10-ish. At around 9pm I was usually back at my place.* |

*Note:* P=participant; R=researcher.

Table 3. Definition and examples for Judgement/estimation stage of the Cognitive model

| **Judgement/estimation** | |
| --- | --- |
| **Definition** | **Examples** |
| Confidence in accuracy, integration of information   - Mental effort to answer accurately (motivation) - Sensitivity/Social desirability | *R: What about palliative care team? How long were they involved in the care?*  *P: Hmmm, I … I can’t … I’m trying to do a memory exercise right now …*    *R: Have you had accumulated debts, had to sell or refinance your house or ask for money as a result of your husband's illness, treatment or death? Yes … no … I don't know or I prefer not to answer.*  *P: I prefer not to answer. It's a difficult part.* |

*Note:* P=participant; R=researcher.

Table 4. Definition and examples for Response stage of the Cognitive model

| **Response processes** | |
| --- | --- |
| **Definition** | **Examples** |
| Matching the internally generated answer to response categories | *R: Now I'm going to ask you to think about this past month. So, this would be August. In the last month, how often have you felt yourself longing or yearning for the person you lost? I have here not at all, at least once, at least once a week, once a day, several times a day.*  *P: I can't quantify it in such a linear way as you are asking me!* |

*Note:* P=participant; R=researcher.

**Analysis by the Feinstein’s Sensibility framework^3^**

Table 5. Descriptive summary of the results of the sensibility analysis with actions for improvement

| **Sensibility aspect^3^** | **Descriptive summary with examples** |
| --- | --- |
| **Overall impression and purpose of the survey**  Relates to comprehensibility and relevance: Is the instrument appropriate for the intended population and purpose.   - Are the survey questions written in plain, accessible language? - Are ambiguous terms clearly defined or explained (e.g. “formal support”)? - Have questions been tested for understanding with bereaved individuals? - Are all questions meaningful and important? - Do items reflect real-world concerns of grieving people? - Are any questions potentially disturbing? | **Summary:**  In general, the survey was well accepted. They described it as comprehensive and did not identify any additional topics or items that were felt as missing. Noone requested to end participation before finishing. One person decided not to participate upon seeing the survey (they requested to see it beforehand) and one person talked to the deceased’s palliative care clinician about the decision to take part in the study. All but one consented to be contacted in the future should this be needed for collection of additional data. Three referred to the interviewer as they would have been a part of the clinical team.  Although slightly distressful, participants had a positive impression of the survey and participation in general. Some highlighted the importance of researching topics such as palliative care, and grief and bereavement since these are less talked about and known topics but “*incredibly important*” if we would like to “*improve well-being and health*”, as well as the services and support.  **Actions:**  Besides measures described in the manuscript, a stronger emphasis will be put on the fact that the researcher is not a part of the clinical team. |
| **Face validity**  Does the survey appear to collect data on what it claims to measure.   - At face value, do the questions appear to address experiences of grief and bereavement support? - Would a bereaved person, clinician, support staff recognise the content as relevant? | **Summary:**  Overall, questionnaire makes sense to the participants. They were able to answer all of the questions, finding them easy and clear to understand. In the analysis of the cognitive response process we identified those items where additional strategies or information is needed to help either with remembering details, making sure the time frame of the question is clear, and help making estimations where there is oscillation in perceived XXX over time (e.g., grief intensity can change over time in both directions, satisfaction with care may vary depending on the team). As one participant described: *“What does “sometimes” or “slightly” mean when you ask me how I feel? Because this oscillates, right? You feel more or less good, depending on the day, depending on what happened on that day. Do you see what I mean? Because as time goes by, the feelings change.* See Table 1-4 for more examples.  The instructions were, in general, clear and easy to understand. Some asked for clarifications in relation to time frame of the question and the person of interest (is the question about the participant or the deceased). One question was too complexed to present over the phone due to many response options and instructions that were too confusing when explained by the researcher. See Tables 1-4 for more examples.  The response categories were adequate, we identified a few questions that need an “not applicable” answer option.  **Actions:**  In addition to the measures identified in the section “Cognitive processes”, we will add introductory text to the grief related questions, acknowledging the fluctuating nature of grief and needs for support and use open ended questions to allow for additional comments and explanation of their experience. |
| **Content validity**  Relates to the extent the survey covers the full scope of the concept being used.   - Does the survey cover full range of relevant experiences (emotional, practical neds, etc.)? - Are there opportunities for participants to share experiences not captured in predefined questions? - Does the survey reflect diversity in bereavement experiences? - Are culturally appropriate constructs and expressions of grief included? | **Summary:**  The survey defines bereavement support in a way that makes sense for the participants and was well understood by them with some minor exceptions, such as whether the survey focuses only on emotional support or are other types of support, e.g. practical, also included (*My lawyer has been very helpful with everything, all the paperwork. Does this count as support as well? Or is it just the emotional part?*).  Grief (PG-13) was for some felt to be described with too intense of a wording and caregiving was not understood to be something burdensome (*I didn’t feel burdened because you do this out of care and love. During that time, you don’t feel exhausted.*) and this was reflected also in quantitative response (see results in the manuscript).  Knowing this was a study about bereavement support, they understood the need to investigate their experience of caregiving and professional care received (sections that some found distressful) and how this was relevant to understand their experience of loss and bereavement support needs.  The survey includes important items that are necessary to describe the need for support and bereavement support accesses and utilised. No repetitive, redundant or missing items were identified. No additional contacts/types of psychosocial bereavement support were identified as missing.  **Actions:**  Identified necessity of a working definition of what entails bereavement support for the purpose of this study, whether by support we understand only emotional and psychological aspects of loss or others, such as practical support. The open-ended questions already included in the survey will be introduced as an additional space to explain and describe their personal experience in more details, with this allowing for the expression of the individuality. |
| **Ease of use**  Relates to feasibility and comprehensibility   - Is the survey length reasonable? - Is it suitable for the intended mode of administration? - Can it be realistically used in research setting? | **Summary:**  Initially, the survey took too long to complete and was found very distressing. The first two survey interviews took around 1.5 hours (more than double than usual) as the first part was about professional care received and the caregiving. Especially the questions about the communication with health care professionals triggered memories, longer narratives and were emotionally burdensome to a point that the researcher suggested a break in one interview.  Language was comprehensible with exception of some PG-13 items (See table above). Questions were easy to understand independently of the level of education which varied (from less than finished primary schooling to masters degree). The question about financial impact of the illness and loss was found sensitive and this was foreseen by the providing “Prefer not to respond” option (used 4 out of 20 times). No other question was identified as too sensitive.  The survey made the participants remember and feel things that were at times intense and required emotional effort to respond, but they found it manageable and felt the researcher was providing supportive environment. See more about experience of participation in section *Impact of the survey and participation*. 20% (13/68) refused to participate (see Figure 1 for more detail).  **Actions:**  Change of the order of the sections. |
| Response options  Relates to appropriateness of response scales. | **Summary:**  Response options were adequate, except for how the PG-13 Likert scale for different manifestations of grief (*not at all, at least once, at least once a week, once a day, several times a day*) where the participants commented that “*it’s not possible to count grief in this way*” or “*you cannot quantify missing someone*”. Some questions were missing “not applicable” option.  **Actions:**  No additional measures taken. |
| Format  Relates to layout, clarity, and structure. | **Summary:**  Overall survey works well. One exception is a question about whether the person felt the need for professional support which then offers 12 different response options capturing also the possible reasons for not having or not accessing support when the support was wish for. The researcher had difficulty presenting this item in a phone interview and resulted in adapting the instructions after one participant stated “*I don’t know how to answer to this question.”*  **Actions:**  The questions was split into parent-child questions that are easy to present independently of the mode of the interview (face-to-face or phone). |
| Episodic component  Relates to the time period of survey administration as well as timeframes for specific items.   - Is the timing of the administration appropriate and clearly justified? - Is time frame of the questions clear and appropriate (can we realistically expect the person will remember)? | **Summary:**  Participants thought the episodic timeframe adequate (e.g.,last three months of life of the deceased), and responses showed that there were no major issues in remembering details (see also T’s table). What proved to be problematic were the changes in the timeframe between the sections/questions (e.g., PG-13 refers to the participant’s last month, while caregiving and professional health care section to last three months of the deceased’s life), with items that at times refer to the experience of the deceased and at times to the experience of the family carer.  Timing of the survey was also welcomed as acceptable *(“The contact comes at the right time because it is neither too close nor to far away from death.”*)  **Actions:**  Signal clearly the change in the time/person focus of the questions. |

**Development of the survey questionnaire**

***Phase 1 - Identifying major topics to be surveyed***

This initial step involved identifying major domains. As survey aimed to examine variations in bereavement support for family carers of cancer patients, we identified the following domains: a) grief and bereavement-related needs to understand what kind of support would be recommended based on the three-tiered model of bereavement support,^4^ b) bereavement support as a response to the individual needs, c) received oncological and palliative care, to capture experiences with healthcare services that may shape the experience of loss^5,6^ and, therefore, be related to access to and utilization of bereavement support, d) caregiving experience and burden as a potential contributors to the need for support into bereavement,^5,6^ e) additional sociodemographic information.

***Phase 2 - Identifying variables within each domain to respond to research question***

Variables were identified based on a systematic review of literature on needs for and access to bereavement support and in consultation with two experts in bereavement support (clinical psychologists with advanced training in grief therapy and more than 15 years of experience in working with the bereaved in public and private sector). The review examined the content and methodology of existing relevant surveys. We also identified factors that could help explain variations in access and potential confounders.

Recognizing that every loss and grieving process is a relational event/process, with a distinctive response to the loss and experienced in a unique way by each bereaved person, we considered carefully how each variable would be reflected and operationalised at an individual, relational and contextual level.

***Phase 3 - Identifying and prioritising items for inclusion in the survey***

We linked each variable identified in the previous step with its potential measure. Previous studies and consultation with experts informed the choice of standardised instruments, choice of items from other studies and formulation of newly developed items. To identify potential difficulties in completing the survey, we anticipated the cognitive tasks required for each item and the potential biases associated with them.

***Phase 4 - Sequencing of the sections and development of instructions***

We anticipated the questions about formal and informal care to be less emotionally burdensome as they involved objective information, such as the duration of professional care, number of hours dedicated to family caregiving, financial hardship. Hence, we placed these questions in the first two sections of the survey. They were followed by a section on the experience of grief and needs for and utilization of bereavement support (expected to be more burdensome for the participant). By this point, ideally, a rapport would be established in the interview between the researcher and the participant. The survey ended with information on socio-demographic variables and questions about the experience with participation in the survey.

***Phase 5 – Consulting with representatives of the bereaved (pre-piloting)***

We simulated the survey interview with two former family carers; one lost the spouse and the other the mother, both due to cancer 3 years prior to our conversation. The interviews lasted 70 and 100 minutes. They provided feedback on the points where the sequence of the items was felt to be disrupted by chronological inconsistencies, which was felt interruptive when recalling information. For both, the questions about communication between the clinical team and the family were found to be most distressing and elicited spontaneous narratives of various events and conversations. They both found this tiring.

The preference of both carers was the phone interview as it gave more privacy, and it was easier to compose themselves again if getting emotional. Both said the interview was distressful, yet a positive experience as they felt not enough was being done for the families after the death, and that the society is not open to talk about grief and mourning as much as the bereaved usually need.

The researcher realized the part about professional care was not working effectively, with lack of alignment between the thematic and temporal sequence of the events the questions were asking about. Time references were found problematic, it was unclear for both carers whether the researcher was asking about events during caregiving or at present. The section about bereavement support was found to be confusing and repetitive (e.g., psychological help was present in two different lists of possible contacts).

***Phase 6 – Constructing the pilot version of the survey***

Based on the previous stage, three items were taken out, one question was added, three were slightly reformulated and reordered, and the order of the sections was changed.

***Phase 7 - Piloting the survey with cognitive interviews***

This phase and its findings are described in the manuscript.

***Phase 8 – Finalizing the survey***

Table 5 shows the final structure of the survey with its sections and number of items. Altogether, the survey contains 79 items (yes/no, multiple choice or short answer questions) and seven open questions. The sections are balanced in terms of number of items.

Table 5. Survey structure for measuring variations in bereavement support at a service level

| **Section** | **Description of items** |
| --- | --- |
| **Introduction** | Short explanation of the aim of the survey. Information on what topics will be covered in the survey, together with a gentle “warning” advising participants that some sections may be more sensitive. Ethical aspects (anonymity, confidentiality, right to not respond to a question or stop participation). Acknowledgement of the uniqueness of the experience of loss and the needs, with questions that do not imply having right or wrong answers. Question whether they would like to suggest the best way to address the deceased (by name, by relationship, etc.). Confirming if there are any doubts or questions before starting. |
| **1 - Bereavement support** | Total: 14 items + 4 open questions |
| Basic information about grief and bereavement support | Receipt of information about grief and available support.  (3 items) |
| Support by family and friends | Felt and observed need for support, received support  (3 items) |
| Specialised formal support | Need for support, help-seeking, barriers, helpfulness, accessibility of support, awareness of existing support, beliefs about professional support .  (7 items)  Open question about help-seeking and barriers |
| Utilization and helpfulness of all levels of support support | 3 items with extensive list of possible contacts and sources of support  Open question about (un)helpfulness of support contacts and activities utilised during the bereavement. |
| **2 - Bereavement support needs** | 18 items |
| Nature of relationship | Kinship, quality of the relationship  (3 items) |
| Signs of prolonged grief | PG-13^7^  13 items |
| Previous losses | Existence of multiple losses and temporal proximity to the current loss  (2 items) |
| **3 - Caregiving experience** | 17 items |
| Objective caregiving burden | Involvement in and intensity of caregiving, additional support, previous experience with caregiving, continuity of support pre- and post-death  (9 items) |
| Subjective caregiving burden | Perceived adequacy of support, intensity of burden  (3 item) |
| Financial hardship and work situation | Work situation (absenteeism) and financial hardship before and after the death, financial burden related to illness and death  (5 items) |
| **4 - Healthcare provided by health care professionals** | 12 items |
| Support received | Duration of care, contact with palliative care  (2 items) |
| Satisfaction with care | General satisfaction and perce3ived adequacy of care provided by clinical teams  (4 items) |
| Symptom management | Management of symptom burden in general  (1 item) |
| Awareness of impending death | Awareness of illness resulting in death  (1 item) |
| Communication with clinical team | Communication about prognosis, adequacy of information provided  (2 items) |
| Presence at the time of death | 1 item |
| Place of death | 1 item |
| **5 - Sociodemographic and clinical information** | 15 items |
| Sociodemographic and clinical information about the deceased person | 4 items |
| Sociodemographic information about the family carer | 9 items |
| Current financial hardship and work situation | Work situation (absentism) and financial hardship at the time of survey completion (2 items) |
| **6 – Final comments and debriefing** | 3 items + open questions |
| Interest in receiving the results | 1 item |
| Experience of participation in the survey | 2 items + 2 open questions |
| Additional comments | Open question |

**References**

1. Tourangeau R. The survey response process from a cognitive viewpoint. *Qual Assur Educ* 2018; 26: 169-181.

2. Tourangeau R. Cognitive aspects of survey design: building a bridge between disciplines. Washington: National Academy Press, 1994.

3. Feinstein AR. The theory and evaluation of sensibility. In: Feinstein AR, ed. Clinimetrics. New Haven: Yale University Press, 1987: 141-166.

4. Aoun SM, Breen LJ, O'Connor M, Rumbold B, Nordstrom C. A public health approach to bereavement support services in palliative care. *Aust J Public Health* 2012; 36: 14-16.

5. Coelho AM, Delalibera MA, Barbosa A. Palliative care caregivers' grief mediators: a prospective study. *Am J Hosp Palliat Care* 2016; 33: 346-353.

6. Lobb EA, Kristjanson LJ, Aoun SM, et al. Predictors of complicated grief: a systematic review of empirical studies. *Death Stud* 2010; 34: 673-698.

7. Prigerson HG, Horowitz MJ, Jacobs SC, et al. Prolonged grief disorder: Psychometric validation of criteria proposed for DSM-V and ICD-11. *PLoS Med* 2009; 6: e1000121-e.

**Survey**

**SUPPORTING FAMILIES IN BEREAVEMENT AFTER CANCER:**

**HOW CAN WE DO BETTER?**

| **INTRODUCTION** |
| --- |

*[Introductory text explaining the aim of the survey, ethical considerations, a brief description of the survey structure, and any other information considered necessary or appropriate to make the survey experience as minimally distressing, and as positive as possible.]*

**PART 1 – BEREAVEMENT SUPPORT**

*[Introductory text]*

| **P1.1** **Did you receive any of the following around or after the death of your relative/friend?** | |
| --- | --- |
| A leaflet or basic information about grief and bereavement in any other form (may include what grief is, common signs of grief, what to expect, etc. | Information about where to look for further support or who to contact if additional support may be needed? |
| 〇 Yes | 〇 Yes |
| ⇒ Did you find this information helpful?  〇 Yes 〇 No | ⇒ Did you find this information helpful?  〇 Yes 〇 No |
| 〇 No | 〇 No |
| 〇No, but I would have liked to | 〇No, but I would have liked to |
| 〇I do not remember | 〇I do not remember |
|  |  |

**P1.2 Who provided you this information?**

| 〇 | Healthcare professional involved in the care of my relative/friend |
| --- | --- |
| 〇 | Healthcare professional not involved in the care of my relative/friend |
| 〇 | I do not know / I do not remember |
| 〇 | Other: ____________________________________ |

**P1.3 Since your [*deceased relative/friend*] died, have you felt the need to talk to someone about topics related to the illness, death, dying and loss?**

| 〇 | Never |
| --- | --- |
| 〇 | Rarely |
| 〇 | Sometimes |
| 〇 | Often |
| 〇 | All the time |

**P1.4 Which of the following apply to your experience with support from family and friends after the death?**

| 〇 | | Family and/or friends have supported me in my grief. |
| --- | --- | --- |
| 〇 | | I did not need their support. |
| 〇 | I was not comfortable asking family and/or friends for support. | |
| 〇 | My family and friends do not have the knowledge or experience to support me. | |
| 〇 | Other: | |

**P1.5 Has any family member, friend, or colleague suggested that it might be helpful for you to talk to someone or seek professional help in relation to your loss?**

| 〇 | Yes |
| --- | --- |
| 〇 | No |

| **P1.6 Since your [*deceased relative/friend*] died, have you felt the need to look for professional support (ex. social worker, psychologist, psychiatrist)?** | | |
| --- | --- | --- |
|  |  |  |
| 〇 | Yes |  |
| 〇 | No |  |
|  |  | |
|  | ⇒ **If Yes**, did you look for professional support?  〇 Yes, and I received/am receiving support.  〇 Yes, but I did not receive any support.  〇 Although I felt the need, I did not look for support. |  |
|  | ⇒ **If Yes** and received support, where did you receive the support?  〇 Public sector services  〇 Private sector services  〇 Other |  |
|  | ⇒ **If you did not look or did not receive support**, please indicate the reason  (select all that apply):  〇 I do not feel comfortable asking for professional support.  〇 I worried I may be judged or misunderstood.  〇 The kind of support I wanted was not available.  〇 I did not know where to look for support.  〇 I was waiting to be contacted by a clinical service.  〇 I could not get in touch with the service.  〇 The waiting time was too long.  〇 I could not afford it.  〇 I could not start due to other practical reasons.  Please specify: ________________________________________________________  〇 I Prefer not to respond.  〇 Other: ______________________________________________________________ |  |
|  | ⇒ **If you did not feel the need** to look for support, what were the reasons?  〇 I received sufficient support from family and/or friends.  〇 I prefer to go through this experience on my own.  〇 Other: _______________________________________________________________ |  |

**P1.7 Please describe any other difficulties or barriers when looking for support, accessing or receiving the support?**

| **P1.8 At any point in your life, have you received psychological support (e.g., from a psychologist, counsellor, mental health professional)?** | |
| --- | --- |
| 〇 | Yes |
| 〇 | No |
| 〇 | I do not know |
|  | |
| **P1.9 Do you know that specialized grief counselling or therapy is available either through the national health system or privately?** | |
| 〇 | Yes |
| 〇 | No |

| **P1.10 In general, do you believe that support from healthcare professionals can help people cope with grief?** | |
| --- | --- |
|  |  |
| 〇 | Strongly disagree |
| 〇 | Disagree |
| 〇 | Neither agree nor disagree |
| 〇 | Agree |
| 〇 | Strongly agree |

| **P1.11 If you needed support, would you prefer to receive the support from the clinical team that cared for your family member/friend during their illness, or would you prefer to look for support from another team or healthcare institution?** | |
| --- | --- |
|  |  |
| 〇 | I would prefer to receive support from the team that cared for my family member/friend. |
| 〇 | I would prefer to look for support from another team or institution. |
| 〇 | I am not sure. |

| **P1.12 In general, how accessible do you think professional support is for people who need it??** | |
| --- | --- |
| 〇 | Very accessible |
| 〇 | Fairly accessible |
| 〇 | Somewhat accessible |
| 〇 | Not very accessible |
| 〇 | Not accessible at all |

**P1.13 In relation to coping with your loss, did you have contact with any of the following people or have you participated in the following activities?**

| **Did you have contact with or participated in ...** | | | |  | **In relation to death, when was this?** |  | **How helpful was this contact/activity in coping with your loss?** | | | | |
| --- | --- | --- | --- | --- | --- | --- | --- | --- | --- | --- | --- |
|  | Yes | No | No, but would have liked to |  | Before (B) or after (A) the death |  | Not at all | Slightly | Moderately | Very | Extremely |
| Family members | 〇 | 〇 | 〇 |  | B/A |  | 〇 | 〇 | 〇 | 〇 | 〇 |
| Friends | 〇 | 〇 | 〇 |  | B/A |  | 〇 | 〇 | 〇 | 〇 | 〇 |
| Work colleagues | 〇 | 〇 | 〇 |  | B/A |  | 〇 | 〇 | 〇 | 〇 | 〇 |
| Funeral home | 〇 | 〇 | 〇 |  | B/A |  | 〇 | 〇 | 〇 | 〇 | 〇 |
| Religious / spiritual leader | 〇 | 〇 | 〇 |  | B/A |  | 〇 | 〇 | 〇 | 〇 | 〇 |
| Hairdresser/Beautician/Barber | 〇 | 〇 | 〇 |  | B/A |  | 〇 | 〇 | 〇 | 〇 | 〇 |
| Social networks (Facebook, Instagram, Twitter) | 〇 | 〇 | 〇 |  | B/A |  | 〇 | 〇 | 〇 | 〇 | 〇 |
| Artificial intelligence AI tools like ChatGPT |  |  |  |  | B/A |  | 〇 | 〇 | 〇 | 〇 | 〇 |
| Reading list or internet resources related to grief and bereavement | 〇 | 〇 | 〇 |  | B/A |  | 〇 | 〇 | 〇 | 〇 | 〇 |
| Public events (Death café, Grief festival) | 〇 | 〇 | 〇 |  | B/A |  | 〇 | 〇 | 〇 | 〇 | 〇 |
| Spiritualist / medium | 〇 | 〇 | 〇 |  | B/A |  | 〇 | 〇 | 〇 | 〇 | 〇 |
| Complementary therapist (yoga, reiki practitioner, acupuncturist, etc.) | 〇 | 〇 | 〇 |  | B/A |  | 〇 | 〇 | 〇 | 〇 | 〇 |
| Legal or financial advisor | 〇 | 〇 | 〇 |  | B/A |  | 〇 | 〇 | 〇 | 〇 | 〇 |
| Pet | 〇 | 〇 | 〇 |  | B/A |  | 〇 | 〇 | 〇 | 〇 | 〇 |
| Support group | 〇 | 〇 | 〇 |  | B/A |  | 〇 | 〇 | 〇 | 〇 | 〇 |
| Activity-based programs specifically designed for bereaved people (nature walks, music, writing/art/movement/dance workshops, etc.) | 〇 | 〇 | 〇 |  | B/A |  | 〇 | 〇 | 〇 | 〇 | 〇 |
| Informal gathering with other bereaved people | 〇 | 〇 | 〇 |  | B/A |  | 〇 | 〇 | 〇 | 〇 | 〇 |
| Other: ___________ | 〇 | 〇 | 〇 |  | B/A |  | 〇 | 〇 | 〇 | 〇 | 〇 |

**P1.14 To help us understand the part these sources have played in your loss, please describe in your own words what you found especially helpful or unhelpful about the support you have had? You can also share if anything changed for you, either for better, for worse, or stayed the same.**

|  |
| --- |

**P1.15 In relation to coping with your loss, did you have contact with any of the following healthcare professionals?**

| **Did you have contact with ...** | | | |  | **In relation to death, when was this?** | |  | **How helpful was this contact in coping with your loss?** | | | | |
| --- | --- | --- | --- | --- | --- | --- | --- | --- | --- | --- | --- | --- |
|  | Yes | No | No, but would have liked to |  | | Before (B) or after (A) the death |  | Not at all | Slightly | Moderately | Very | Extremely |
| Psychologist on your relative’s/friend’s healthcare team | 〇 | 〇 | 〇 |  | | B/A |  | 〇 | 〇 | 〇 | 〇 | 〇 |
| Social worker on your relative’s/friend’s healthcare team | 〇 | 〇 | 〇 |  | | B/A |  | 〇 | 〇 | 〇 | 〇 | 〇 |
| Nurse on your relative’s/friend’s healthcare team | 〇 | 〇 | 〇 |  | | B/A |  | 〇 | 〇 | 〇 | 〇 | 〇 |
| Medical doctor on your relative’s/friend’s healthcare team | 〇 | 〇 | 〇 |  | | B/A |  | 〇 | 〇 | 〇 | 〇 | 〇 |
| Professional for nursing/care home | 〇 | 〇 | 〇 |  | | B/A |  | 〇 | 〇 | 〇 | 〇 | 〇 |
| Family doctor | 〇 | 〇 | 〇 |  | | B/A |  | 〇 | 〇 | 〇 | 〇 | 〇 |
| Pharmacist | 〇 | 〇 | 〇 |  | | B/A |  | 〇 | 〇 | 〇 | 〇 | 〇 |
| School psychologist / social worker  [if you have children] |  |  |  |  | | B/A |  | 〇 | 〇 | 〇 | 〇 | 〇 |
| Social worker (not from your relative’s/friend’s healthcare team) | 〇 | 〇 | 〇 |  | | B/A |  | 〇 | 〇 | 〇 | 〇 | 〇 |
| Psychologist (not from your relative’s/friend’s healthcare team) | 〇 | 〇 | 〇 |  | | B/A |  | 〇 | 〇 | 〇 | 〇 | 〇 |
| Psychiatrist (not from your relative’s/friend’s healthcare team) | 〇 | 〇 | 〇 |  | | B/A |  | 〇 | 〇 | 〇 | 〇 | 〇 |
| Other healthcare professional (not from your relative’s/friend’s healthcare team) | 〇 | 〇 | 〇 |  | | B/A |  | 〇 | 〇 | 〇 | 〇 | 〇 |
| Medication | 〇 | 〇 | 〇 |  | | B/A |  | 〇 | 〇 | 〇 | 〇 | 〇 |
| Other 1: ____________________ | 〇 | 〇 | 〇 |  | | B/A |  | 〇 | 〇 | 〇 | 〇 | 〇 |
| Other 2: ____________________ | 〇 | 〇 | 〇 |  | | B/A |  | 〇 | 〇 | 〇 | 〇 | 〇 |

**P1.16 To help us understand the part these sources have played in your loss, please describe in your own words what you found especially helpful or unhelpful about the support you have had? You can also share if anything changed for you, either for better, for worse, or stayed the same.**

|  |
| --- |

**P1.17 After your relative/friend died, were any of the following offered to you or made available?**

| **Bereavement follow-up care** | | | | |  | | **How helpful was this for coping with your loss?** | | | | |
| --- | --- | --- | --- | --- | --- | --- | --- | --- | --- | --- | --- |
|  | Yes | No | No, but would have liked to |  | | Not at all | | Slightly | Moderately | Very | Extremely |
| Time alone with the deceased after death or a quiet room to be alone after the death | 〇 | 〇 | 〇 |  | | 〇 | | 〇 | 〇 | 〇 | 〇 |
| A condolence letter sent by the healthcare team | 〇 | 〇 | 〇 |  | | 〇 | | 〇 | 〇 | 〇 | 〇 |
| Phone call/email contact from the healthcare after the death | 〇 | 〇 | 〇 |  | | 〇 | | 〇 | 〇 | 〇 | 〇 |
| Home visit from the team after the death | 〇 | 〇 | 〇 |  | | 〇 | | 〇 | 〇 | 〇 | 〇 |
| Members of the healthcare team were present at the wake and/or funeral | 〇 | 〇 | 〇 |  | | 〇 | | 〇 | 〇 | 〇 | 〇 |
| A card or letter sent by the healthcare team around the on-year anniversary of death | 〇 | 〇 | 〇 |  | | 〇 | | 〇 | 〇 | 〇 | 〇 |
| Outro 1: ____________________ | 〇 | 〇 | 〇 |  | | 〇 | | 〇 | 〇 | 〇 | 〇 |
| Outro 2: ____________________ | 〇 | 〇 | 〇 |  | | 〇 | | 〇 | 〇 | 〇 | 〇 |

**P1.18 To help us understand the part these sources have played in your loss, please describe in your own words what you found especially helpful or unhelpful about the support you have had? You can also share if anything changed for you, either for better, for worse, or stayed the same.**

|  |
| --- |

**PART 2 – EXPERIENCE OF LOSS**

*[Introductory text]*

**P2.1 The person who died was your:**

| 〇 | Wife or husband/partner | 〇 | Sibling | |
| --- | --- | --- | --- | --- |
| 〇 | Daughter or son | 〇 | Granddaughter/son | |
| 〇 | Stepdaughter or stepson | 〇 | Other family member (please specify) | |
| 〇 | Parent |  | __________________________ | |
| 〇 | Mother/father-in-law | 〇 | Other (please specify) |  |
| 〇 | Daughter/son-in-law |  | __________________________ | |

**P2.2 How emotionally close did you feel to [*deceased family member/friend*]?**

| 〇 | Not at all close |
| --- | --- |
| 〇 | Slightly close |
| 〇 | Somewhat close |
| 〇 | Quite close |
| 〇 | Very close |
| 〇 | Prefer not to say |

**P2.3 Would you describe your relationship with [*deceased family member/friend*] as difficult or conflictuous?**

| 〇 | Yes |
| --- | --- |
| 〇 | No |
| 〇 | Prefer not to say |

**P2.4-16 PG-13 Items**

**P2.17 Have you experienced the loss of someone important to you in the past?**

| 〇 | Yes |
| --- | --- |
| 〇 | No |

**P2.18 Did the loss(es) occur between one to three years ago?**

| 〇 | Yes |
| --- | --- |
| 〇 | No |

**PART 3 – EXPERIENCE OF LOOKING AFTER OR HELP TAKING CARE**

*[Introductory text]*

**P3.1 Did you look after or help take care of [*deceased family member/friend*] in the last three months before they died?**

| 〇 | Yes |
| --- | --- |
| 〇 | No |

**P3.2 In total, how long did you look after or help take care for [*deceased family member/friend*]?**_________ [months]

**P3.3 How many hours per week did you look after or help take care for [*deceased family member/friend*]?**_________ [hours]

**P3.4 Did other friends or family members look after or help take care of [*deceased family member/friend*]?**

| 〇 | Yes |
| --- | --- |
| 〇 | No |

**P3.5 If yes, how many other friends and family members helped?** _______

**P3.6 Did you receive help from healthcare professionals, care aides, volunteers or other home-based services in looking after or helping take care of [*deceased family member/friend*]?**

| 〇 | Yes |
| --- | --- |
| 〇 | No |

**P3.7 How many other people helped?** _______

**P3.8 Was this the first time you looked after or helped take care of someone with a serious or advanced illness?**

| 〇 | Yes |
| --- | --- |
| 〇 | No |

**P3.9 Thinking back to the time you were looking after or help take care, did you feel you had as much practical help and support as needed?**

| 〇 | Yes |
| --- | --- |
| 〇 | Some, but not as much as needed |
| 〇 | No |

**P3.10 Thinking back to the time you were looking after or help take care, did you feel you had as much emotional support as needed?**

| 〇 | Yes |
| --- | --- |
| 〇 | Some, but not as much as needed |
| 〇 | No |

**P3.11 Thinking back to the time you were looking after or help take care, how would you rate overall burden of your caregiving responsibilities?**

| 〇 | No burden |
| --- | --- |
| 〇 | Slight burden |
| 〇 | Some burden |
| 〇 | High burden |
| 〇 | Extreme burden |

**P3.12 Did any of the people who looked after, helped take care or provided healthcare also supported you during your bereavement?**

| 〇 | Yes |
| --- | --- |
| 〇 | No |

**P3.13 Did you stop working or reduced work because of your [*deceased family member/friend*] illness in the last 3 months before they died?**

| 〇 | Yes |
| --- | --- |
| 〇 | Yes, I carried on working, but with reduced hours |
| 〇 | Yes, I was on annual leave, sick leave, caregiving leave, or in any other form of temporary work interruption |
| 〇 | No, I am permanently sick or disabled |
| 〇 | No, I carried on working equal hours |
| 〇 | No, I was unemployed |
| 〇 | No, I was retired |
| 〇 | No, I was studying |
| 〇 | No, I look after the home |
| 〇 | Other situation (Please specify): ___________________________________ |

**P3.14 What about in the first three months after [*deceased family member/friend*] died, did you stop working or reduce work due to [*deceased family member/friend*] death?**

| 〇 | Yes |
| --- | --- |
| 〇 | Yes, I carried on working, but with reduced hours |
| 〇 | Yes, I was on annual leave, sick leave, caregiving leave, or in any other form of temporary work interruption |
| 〇 | No, I carried on working equal hours |
| 〇 | No, I am permanently sick or disabled |
| 〇 | No, I was unemployed |
| 〇 | No, I was retired |
| 〇 | No, I was studying |
| 〇 | No, I look after the home |
| 〇 | Other situation (Please specify): ___________________________________ |

**P3.15 During the time you were looking after or help take care of [*deceased family member/friend*], which of the following descriptions comes closest to how you feel about your household’s income?**

| 〇 | Living comfortably on the household’s income at the time |
| --- | --- |
| 〇 | Coping on the household’s income at the time |
| 〇 | Difficult on the household’s income at the time |
| 〇 | Very difficult on the household’s income at the time |

**P3.16** **Thinking on the time after the death of [*deceased family member/friend*], which of the following descriptions comes closest to how you feel about your household’s income?**

| 〇 | Living comfortably on the household’s income at the time |
| --- | --- |
| 〇 | Coping on the household’s income at the time |
| 〇 | Difficult on the household’s income at the time |
| 〇 | Very difficult on the household’s income at the time |

**P3.17 Did you accumulate debt, sell or refinance your home, or needed to ask friends or family for money because of the illness and/or death?**

| 〇 | Yes |
| --- | --- |
| 〇 | No |
| 〇 | I do not know |
| 〇 | Prefer not to respond |

**PART 4 – EXPERIENCE OF RECEIVED HEALTHCARE**

*[Introductory text]*

**P4.1 How long did you receive support from the following healthcare teams?**

Oncology: ________

Palliative care: ________

〇 [*Deceased family member/friend*] was referred to palliative care but did not receive it.

〇 [*Deceased family member/friend*] was not referred to palliative care.

**P4.2 If palliative care team was involved in the care of [*deceased family member/friend*], please specify the type:**

| 〇 | No contact with palliative care |
| --- | --- |
| 〇 | Outpatient clinic |
| 〇 | Inpatient hospital palliative care team |
| 〇 | Palliative care unit |
| 〇 | Community palliative care team |

**P4.3 Do you think [*deceased family member/friend*] got as much help as needed from the oncology team?**

| 〇 | Yes |
| --- | --- |
| 〇 | Some help, but not as much as needed |
| 〇 | No |

**P4.4 Overall, do you feel that the care [*deceased family member/friend*] got from oncology team was:**

| 〇 | Excellent |
| --- | --- |
| 〇 | Very good |
| 〇 | Good |
| 〇 | Fair |
| 〇 | Poor |
| 〇 | Very poor |

⇒ *If you received care from palliative care team:*

**P4.5 Do you think [*deceased family member/friend*] got as much help as needed from the palliative care team?**

| 〇 | Yes |
| --- | --- |
| 〇 | Some help, but not as much as needed |
| 〇 | No |

⇒ *If you received care from palliative care team:*

**P4.6** **Overall, do you feel that the care [*deceased family member/friend*] got from palliative care team was:**

| 〇 | Excellent |
| --- | --- |
| 〇 | Very good |
| 〇 | Good |
| 〇 | Fair |
| 〇 | Poor |
| 〇 | Very poor |

**P4.7 Do you feel that the healthcare professionals did enough to help relieve any symptoms [*deceased family member/friend*] had (e.g. pain, nausea/vomiting, diarrhoea, breathlessness, anxiety/depression)?**

| 〇 | Yes, I feel the healthcare professionals did as much as possible |
| --- | --- |
| 〇 | No, I feel the healthcare professionals could have done more |
| 〇 | Family member/friend did not have any symptoms |

**P4.8 Did you know your [*deceased family member/friend*] was likely to die because of the illness?**

| 〇 | Yes |
| --- | --- |
| 〇 | No |

**P4.9 Did any health professional discuss with you, other family members or friends the fact that your [*deceased family member/friend*] was likely to die because of the illness?**

| 〇 | Yes |
| --- | --- |
| 〇 | No |

**P4.10 The information about the diagnosis, prognosis or death that was discussed with you, other family members or friends was:**

| 〇 | Not sufficient |
| --- | --- |
| 〇 | Sufficient |
| 〇 | Too much information |

**P4.11 Were you with your family member/friend when they died?**

| 〇 | Yes |
| --- | --- |
| 〇 | No |

**P4.12 Where did your family member/friend die?**

| 〇 | At home |
| --- | --- |
| 〇 | In hospital – in a palliative care unit |
| 〇 | In hospital – in the emergency department |
| 〇 | In hospital – other |
| 〇 | In a nursing/residential home |
| 〇 | Elsewhere (please specify): |

**PART 5 – ADDITIONAL INFORMATION**

*[Introductory text]*

**P5.1 How old was your family member/friend when they died? _________**

**P5.2 Date of death: _________**

**P5.3 Type of cancer:**

| **Primary site:** | | **Metastases:** | | |
| --- | --- | --- | --- | --- |
| 〇 | Lung | 〇 | Lung |  |
| 〇 | Breast | 〇 | Liver |  |
| 〇 | Stomach | 〇 | Bones |  |
| 〇 | Oesophagus | 〇 | Lymph nodes |  |
| 〇 | Pancreas | 〇 | Central Nervous System (CNS) |  |
| 〇 | Uterus | 〇 | No metastases |  |
| 〇 | Central Nervous System | 〇 | Unknown |  |
| 〇 | Skin | 〇 | Other (please specify): |  |
| 〇 | Prostate |  |  |  |
| 〇 | Colorectal |  |  |  |
| 〇 | Cervix/Cervical cancer |  |  |  |
| 〇 | Bladder |  |  |  |
| 〇 | Haematologic cancers  (lymphoma, leukaemia, multiple myeloma) |  |  |  |
| 〇 | Head and neck |  |  |  |
| 〇 | Unknown |  |  |  |
| 〇 | Other (please specify): |  |  |  |

**P5.4 What best describes [*deceased family member/friend*] gender?**

| 〇 | Female |
| --- | --- |
| 〇 | Male |
| 〇 | Non-binary |
| 〇 | Prefer to self-describe: _________________________ |
| 〇 | Prefer not to say |

**P5.5 How old are you? _______ [or date of birth: ____ / ____ /** ________]

**P5.6 What best describes your gender?**

| 〇 | Female |
| --- | --- |
| 〇 | Male |
| 〇 | Non-binary |
| 〇 | Prefer to self-describe: _________________________ |
| 〇 | Prefer not to say |

**P5.7 In which country were you born?**

[list of countries]

**P5.8 In which country was your mother born?**

[list of countries]

**P5.9 In which country was your father born?**

[list of countries]

**P5.10 Do you have any religious or spiritual beliefs?**

| 〇 | Yes |
| --- | --- |
| 〇 | No |
| 〇 | Prefer not to say |

**P5.11 If yes, which of the following options describes best your beliefs?**

| 〇 | Agnostic |
| --- | --- |
| 〇 | Spiritual but not religious |
| 〇 | Christianity (all denominations) |
| 〇 | Buddhism |
| 〇 | Hinduism |
| 〇 | Islam |
| 〇 | Judaism |
| 〇 | Other |
|  |  |

**P5.12 Which of the following descriptions comes closest to how you feel about your current household’s income?**

| 〇 | Living comfortably on the household’s income |
| --- | --- |
| 〇 | Coping on the household’s income |
| 〇 | Difficult on the household’s income |
| 〇 | Very difficult on the household’s income |

**P5.13 What is your marital status?**

| 〇 | Single |
| --- | --- |
| 〇 | Married/In a civil partnership |
| 〇 | Divorced |
| 〇 | Separated |
| 〇 | Widowed |
| 〇 | Prefer not to say |

**P5.14 What is the highest level of education completed?**

[*response options adapted to specific country*]

**P5.15 What is your current employment status?**

| 〇 | Working |
| --- | --- |
| 〇 | On annual leave, sick leave, unpaid leave or on other temporary work leave situation |
| 〇 | Unemployed |
| 〇 | Permanently sick or disabled |
| 〇 | Retired |
| 〇 | Student |
| 〇 | Looking after the home |
| 〇 | Other (please specify): |

**FINAL COMMENTS**

If there is anything else you would like to tell us about any aspect of your experience of loss, the support you have received or support you would have liked to receive, please use the blank space below.

|  |
| --- |

**EXPERIENCE OF THE QUESTIONNAIRE**

**EXP1 How upsetting was it to answer to this questionnaire?**

| 〇 | Extremely upsetting |
| --- | --- |
| 〇 | Very upsetting |
| 〇 | Somewhat upsetting |
| 〇 | Slightly upsetting |
| 〇 | Not upsetting at all |

**EXP2 If you found it upsetting to answer to this questionnaire, which part was the most upsetting for you? Please write down in the space below, this will help us understand and improve future questionnaires.**

**EXP3 Did you find this questionnaire helpful?**

| 〇 | Extremely helpful |
| --- | --- |
| 〇 | Very helpful |
| 〇 | Somewhat helpful |
| 〇 | Slightly helpful |
| 〇 | Not helpful at all |

**EXP4 If you found it helpful to answer to this questionnaire, please tell us what part was helpful to you and why. This will help us understand and improve future questionnaires.**

|  |
| --- |

*[Closing text]*
